# Correction: The Secreted Triose Phosphate Isomerase of *Brugia malayi* Is Required to Sustain Microfilaria Production *In Vivo*

**DOI:** 10.1371/journal.ppat.1004133

**Published:** 2014-04-28

**Authors:** The PLOS Pathogens Staff

## Notice of Republication

This article was republished on April 2, 2014, to correct Roman letters that were substituted for Greek letters in the Introduction, Results, Discussion, Materials and Methods, and Figure 2 Legend. Please download this article again to view the correct version. The originally published, uncorrected article and the republished, corrected article are provided here for reference.

## Supporting Information

File S1 Originally published, uncorrected article.
(PDF)

File S2 Republished, corrected article.
(PDF)
