# Supplementary figures and images for: The Secreted Triose Phosphate Isomerase of Brugia malayi Is Required to Sustain Microfilaria Production In Vivo
Source: PLoS Pathog. 2014 Feb 27;10(2):e1003930. doi: 10.1371/journal.ppat.1003930 (PMC3937304; doi:10.1371/journal.ppat.1003930)

## Hewitson *et al.*, Figure S 1

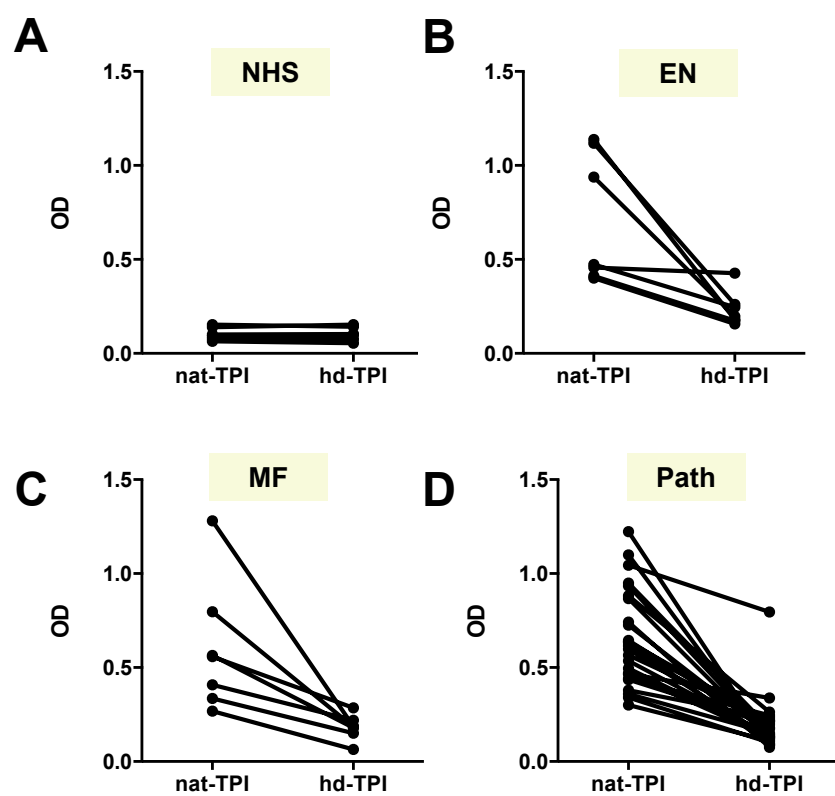

Supplement: Figure S1 — Human antibodies to Bm-TPI recognise conformational epitopes. IgG1 human ELISA reactivity to “native” or heat-denatured recombinant Bm-TPI. Sera is from (A) non-exposed UK residents, (B) endemic normals, (C) microfilaremics, and (D) pathology patients. Only sera producing a positive signal were included in B–D. (PDF) [file ppat.1003930.s001.pdf]

## Hewitson *et al.*, Figure S 2

**A** MOPC31C

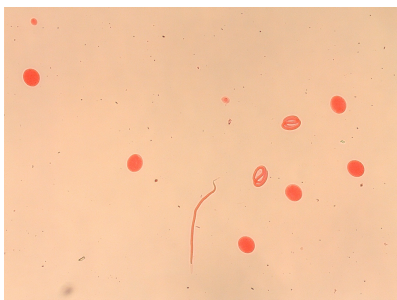

**B** anti-*Bm*-TPI

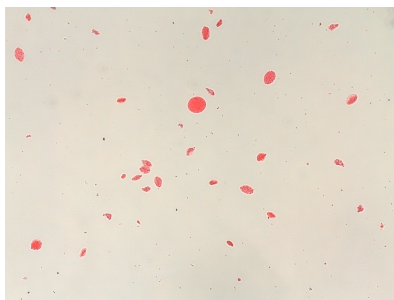

Supplement: Figure S2 — Bm-TPI neutralisation impairs embryogenesis. Representative pictures of uterine contents of adult female B. malayi parasites recovered from (A) control MOPC31C and (B) 1.11.1 anti-TPI antibody treated mice after 14 days. Note oocytes in control worms with pretzel and stretched Mf, whereas uterine contents in anti-Bm-TPI worms are smaller and potentially degraded. (PDF) [file ppat.1003930.s002.pdf]

# Hewitson *et al.*, Figure S 3

**A**

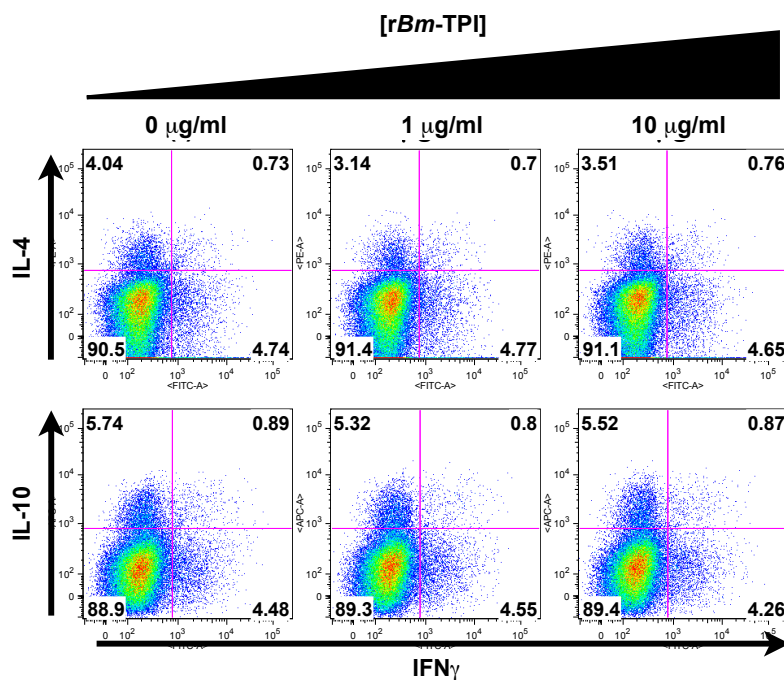

**B**

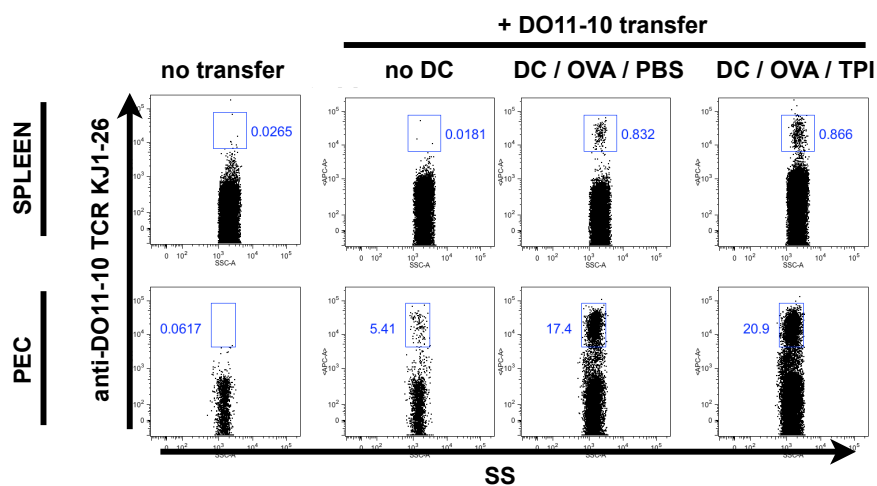

**C**

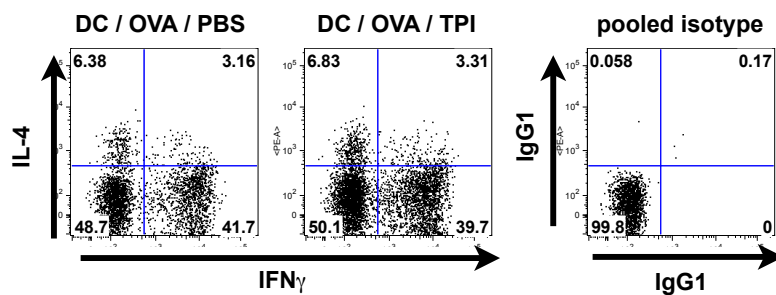

Supplement: Figure S3 — In vitro or in vivo exposure to Bm -TPI does not alter T cell responses. A. In vitro production of IL-4, IL-10 and IFN-γ by anti-CD3/anti-CD28 stimulated CD4+ T cells ± Bm-TPI. B. In vivo transfer of OVA-specific DO11.10 transgenic T cells with pOVA-pulsed BMDC ± Bm-TPI. (B) Percentage of spleen (top) or peritoneal (bottom) CD4+ that are OVA-specific. C. Ex vivo production of IFN-γ and IL-4 by gated peritoneal KJ1-26+CD4+ cells. Similar results to (B–C) were observed if mice were primed with OVA protein adsorbed to alum adjuvant rather than DC (data not shown). (PDF) [file ppat.1003930.s003.pdf]
