# Supplementary material for: Correction: The Secreted Triose Phosphate Isomerase of Brugia malayi Is Required to Sustain Microfilaria Production In Vivo
Source: PLoS Pathog. 2014 Apr 28;10(4):e1004133. doi: 10.1371/journal.ppat.1004133 (PMC4002453; doi:10.1371/journal.ppat.1004133)
Supplement: File S2 — Republished, corrected article. (PDF) [file ppat.1004133.s002.pdf]

OPEN ACCESS Freely available online

PLOS | PATHOGENS

The Secreted Triose Phosphate Isomerase of *Brugia malayi* Is Required to Sustain Microfilaria Production *In Vivo*

James P. Hewitson[1], Dominik Rückerl[1], Yvonne Harcus[1], Janice Murray[1], Lauren M. Webb[1], Simon A. Babayan[1¤], Judith E. Allen[1], Agnes Kurniawan[2], Rick M. Maizels[1]*

1 Institute of Immunology and Infection Research, University of Edinburgh, Edinburgh, United Kingdom, 2 Department of Parasitology, University of Indonesia, Jakarta, Indonesia

Abstract

Human lymphatic filariasis is a major tropical disease transmitted through mosquito vectors which take up microfilarial larvae from the blood of infected subjects. Microfilariae are produced by long-lived adult parasites, which also release a suite of excretory-secretory products that have recently been subject to in-depth proteomic analysis. Surprisingly, the most abundant secreted protein of adult *Brugia malay*i is triose phosphate isomerase (TPI), a glycolytic enzyme usually associated with the cytosol. We now show that while TPI is a prominent target of the antibody response to infection, there is little antibody-mediated inhibition of catalytic activity by polyclonal sera. We generated a panel of twenty-three anti-TPI monoclonal antibodies and found only two were able to block TPI enzymatic activity. Immunisation of jirds with *B. malayi* TPI, or mice with the homologous protein from the rodent filaria *Litomosoides sigmodontis*, failed to induce neutralising antibodies or protective immunity. In contrast, passive transfer of neutralising monoclonal antibody to mice prior to implantation with adult *B. malayi* resulted in 60–70% reductions in microfilarial levels *in vivo* and both oocyte and microfilarial production by individual adult females. The loss of fecundity was accompanied by reduced IFNγ expression by $CD4^+$ T cells and a higher proportion of macrophages at the site of infection. Thus, enzymatically active TPI plays an important role in the transmission cycle of *B. malayi* filarial parasites and is identified as a potential target for immunological and pharmacological intervention against filarial infections.

Citation: Hewitson JP, Rückerl D, Harcus Y, Murray J, Webb LM, et al. (2014) The Secreted Triose Phosphate Isomerase of *Brugia malayi* Is Required to Sustain Microfilaria Production *In Vivo*. PLoS Pathog 10(2): e1003930. doi:10.1371/journal.ppat.1003930

Editor: Edward Mitre, Uniformed Services University of the Health Sciences, United States of America

Received July 29, 2013; **Accepted** January 2, 2014; **Published** February 27, 2014

Copyright: © 2014 Hewitson et al. This is an open-access article distributed under the terms of the Creative Commons Attribution License, which permits unrestricted use, distribution, and reproduction in any medium, provided the original author and source are credited.

Funding: JPH, YH, JM and RMM are funded by the Wellcome Trust (Ref 090281), LM by Medical Research Council UK studentship, SB by the EU FP7 grant "EPIAF" (no 242131), DR and JEA by the Medical Research Council UK (G0600818). The funders had no role in study design, data collection and analysis, decision to publish, or preparation of the manuscript.

Competing Interests: The authors have declared that no competing interests exist.

* E-mail: r.maizels@ed.ac.uk

¤ Current address: Institute of Biodiversity, Animal Health & Comparative Medicine, University of Glasgow, Glasgow, United Kingdom.

Introduction

Continued survival of parasitic helminths within their mammalian host requires that they neutralise potentially protective immune responses, generate energy and reproduce. Filarial nematodes are particularly long-lived, tissue-dwelling parasites which evade immunity and maintain transmission over many years [1]. Over 100 million people are infected with lymphatic filariae, such as *Brugia malayi*, and no vaccine is available for human use [2,3]. Transmission occurs when blood-borne microfilarial larvae are taken up by a mosquito vector, generating infective third-stage larvae which enter humans on a subsequent blood-meal. Hence, any immunological means of blocking microfilarial release would interrupt transmission.

As extracellular pathogens, the interaction of live parasites with both the host and each other is likely to occur through a combination of excretory/secretory (ES) products and surface molecules [4,5]. Given the presumed involvement of ES molecules in a range of processes essential for successful parasitism, they represent attractive vaccine and drug targets. Because of this, we and others have taken a proteomic approach to characterise the complex mixture of proteins secreted by the human filarial nematode *Brugia malayi* (*B. malayi* ES, BES) [6–9]. This revealed that the most abundant ES protein of adult *B. malayi* is the glycolytic enzyme triose phosphate isomerase (*Bm*-TPI, EC 5.3.1.1), predominantly from female worms. Detailed analysis of the secretions of all life cycle stages has revealed that TPI is also released by moulting L3 larvae early in infection [8].

TPI catalyses the interconversion of the triose phosphates glyceraldehyde 3-phosphate and dihydroxyacetone phosphate, an essential step in glycolysis and gluconeogenesis [10]. Whilst TPI has been detected in the ES products of other worms, such as the cercariae and eggs of *Schistosoma mansoni* [11,12] and adult *Haemonchus contortus* [13], the levels appear low compared to the large amounts released by adult *B. malayi* [6–8]. Furthermore, there is little or no secretion of other glycolytic enzymes, implying that TPI is selectively secreted through an active process, rather than simply leaching from compromised worms. This was supported by the demonstration that TPI is approximately 20-fold enriched in BES compared to a soluble extract of adult *B. malayi* [6].

Author Summary

Triose phosphate isomerase (TPI) is a ubiquitous and highly conserved enzyme in intracellular glucose metabolism. Surprisingly, the human lymphatic filariai nematode parasite *Brugia malayi*, releases TPI into the extracellular environment, suggesting a role in helminth survival in the mammalian host. We first established that *B. malayi-infected* humans and rodents generate TPI-specific serum antibody responses, confirming presentation of this protein to the host immune system. However, immunisation of rodents with *B. malayi* TPI did not induce protection against infection. Furthermore, TPI from a related parasite, *Litomosoides sigmodontis*, did not induce protective immunity in mice. Notably, antibodies from infected hosts did not neutralise the enzymatic activity of TPI. We then generated twenty-three anti-TPI monoclonal antibodies, of which only two inhibited enzymatic activity. Transfer of neutralising antibody to mice prior to *B. malayi* infection effected a 69.5% reduction in microfilarial levels *in vivo* and a 60% reduction in microfilariae produced by individual adult female parasites. Corresponding shifts in the host immune response included reduced Th1 cytokine production and enhanced macrophage numbers. Enzymatically active TPI therefore promotes production of the transmission stage of *B. malayi* filarial parasites and represents a rational target for new vaccine and drug development to protect against filarial infections.

However, it is unclear why *Brugia* and other nematodes secrete TPI given that glycolysis occurs in the cytosol. In this regard, several reports indicate that TPI is a multifunctional protein. For instance, TPI binds to the intracellular tail of the integrin αIIb in platelets and may regulate integrin signalling [14]. TPI can also function as an extracellular adherence molecule, and in this way mediates the interaction of the fungal pathogen *Paracoccidiodes brasiliensis* with both host epithelial cells and the extracellular matrix proteins laminin and fibronectin [15]. Similarly, surface associated TPI of *Staphylococcus aureus* contains a lectin activity that can bind fungal sugars and promote bacterial adherence to, and subsequent killing of, *Cryptococcus neoformans* [16,17]. Additionally, studies of human TPI deficiency have shown that exogenous TPI can complement TPI-deficient cells, suggesting that the secreted enzyme may be taken up in a functional form by surrounding cells [18,19].

Glycolytic enzymes, including TPI, have also been tested as targets of protective immunity. Thus, a neutralizing monoclonal antibody to *S. mansoni* TPI can confer up 40–50% reduction in worm burden in mice [20] and DNA vaccination with *S. japonicum* TPI reduces worm and egg burdens in experimentally infected pigs and water buffalo [21,22]. Successful vaccination with schistosome TPI is consistent with its induction of IFNγ, a cytokine associated with protective immunity against the larval schistosomula [23]. Even in the Th2-dominated environment that develops following schistosome egg production in mice, TPI preferentially stimulates Th1 cytokines [24]. *S. mansoni* TPI can also induce Th1 differentiation by T cells from unexposed humans [25]. In other helminth species, certain glycolytic enzymes have been similarly tested as vaccine candidates: for example, *Onchocerca volvulus* fructose 1,6-biphosphate aldolase is strongly recognised by antibodies from exposed but uninfected subjects, and can elicit a 50% reduction in larval survival in vaccinated mice [26]. However, studies on *B. malayi* have to date focussed solely on the biochemical properties of glycolytic enzymes with a view to development of new pharmacological inhibitors [27,28].

Since glycolysis plays a key role in filarial worm energy metabolism [29–31], coupled with the unusually high level of secretion of TPI, we investigated the role of *Bm*-TPI in *B. malayi* infection. We confirmed that *Bm*-TPI is highly preferentially secreted, enzymatically active, and an antibody target in both infected mice and humans. Whilst vaccination with filarial TPI failed to confer protection against challenge infection with *B. malayi or Litomosoides sigmodontis*, antibody-mediated neutralisation of *Bm*-TPI shows it is required for the optimal survival of microfilariae within the mammalian host. As such, this parasite enzyme represents a novel and rational target for intervention by immunological or pharmacological means.

Results

Cloning and characterisation of recombinant *Bm*-TPI and *Ls*-TPI

To study the role of filarial triose phosphate isomerases, we first cloned the cDNA encoding this enzyme for expression as recombinant proteins. Full-length *Bm*-TPI cDNA was amplified by PCR from mixed adult *B. malayi* cDNA, cloned and confirmed as identical to the annotated *B. malayi* gene (*Bm*1_29130 [32]). The encoded 247-aa protein lacks a signal sequence, and has a predicted molecular weight of 27,097 Da. Sequence analysis shows a high degree of amino acid conservation with human (61% identity), *S. mansoni* (58%) and *C. elegans* (76%) proteins, including the AYEPVWAIGTG active loop and catalytic E165 (corresponding to E166 in human TPI; [33]), as well as the other active site residues, N10 (human N12), K12 (K14) and H94 (H96) (**Fig. 1 A**).

Recombinant *Bm*-TPI was expressed in bacteria and purified by nickel resin affinity chromatography, appearing as a single band of approx. 28 kDa by SDS-PAGE and a dominant molecular species by mass spectrometry of 28,030 (data not shown). Functional activity of recombinant *Bm*-TPI was confirmed by enzymatic assay, in which it displayed typical Michaelis-Menten kinetics indistinguishable from rabbit TPI with a Vmax of 715 U/mg and a Km of 1.8 mM (**Fig. 1 B**). The activity of *Bm*-TPI was compared to the homologous enzyme from the mouse filarial parasite *Litomosoides sigmodontis* [34]. *Ls*-TPI has 94% (233/247) amino acid identity to *Bm*-TPI (**Fig. 1A**), and following cloning of the corresponding cDNA and bacterial expression, recombinant protein showed similar enzyme kinetics to both *Bm*-TPI and rabbit TPI (**Fig. 1 B**).

Abundant expression of *Bm*-TPI

Previous proteomic studies have indicated *Bm*-TPI is amongst the most abundant proteins secreted by adult female *B. malayi* [6–8]. Preferential secretion was confirmed by Western blot using a polyclonal antiserum raised against r*Bm*-TPI, which showed significant enrichment of *Bm*-TPI in BES compared to somatic extracts of adult worms, L3 larvae and microfilariae (**Fig. 2 A**). Native secreted *Bm*-TPI was shown to be enzymatically active by comparing BES with varying amounts of recombinant *Bm*-TPI. This revealed that each μg of BES had equivalent enzymatic activity to 370±86 ng recombinant protein (**Fig. 2 B**). Enzymatic activity in BES was abolished by heat denaturation (**Fig. 2 B**).

Immunohistochemistry of sections of adult male and female *B. malayi* showed ubiquitous somatic expression pattern expected of a glycolytic enzyme, but provided no clues as to the source of secreted *Bm*-TPI by adult females (**Fig. 2 C**). Additionally, surface staining of intact whole worms was not seen, indicating that *Bm*-TPI is not shed from the cuticle of the parasite (data not shown).

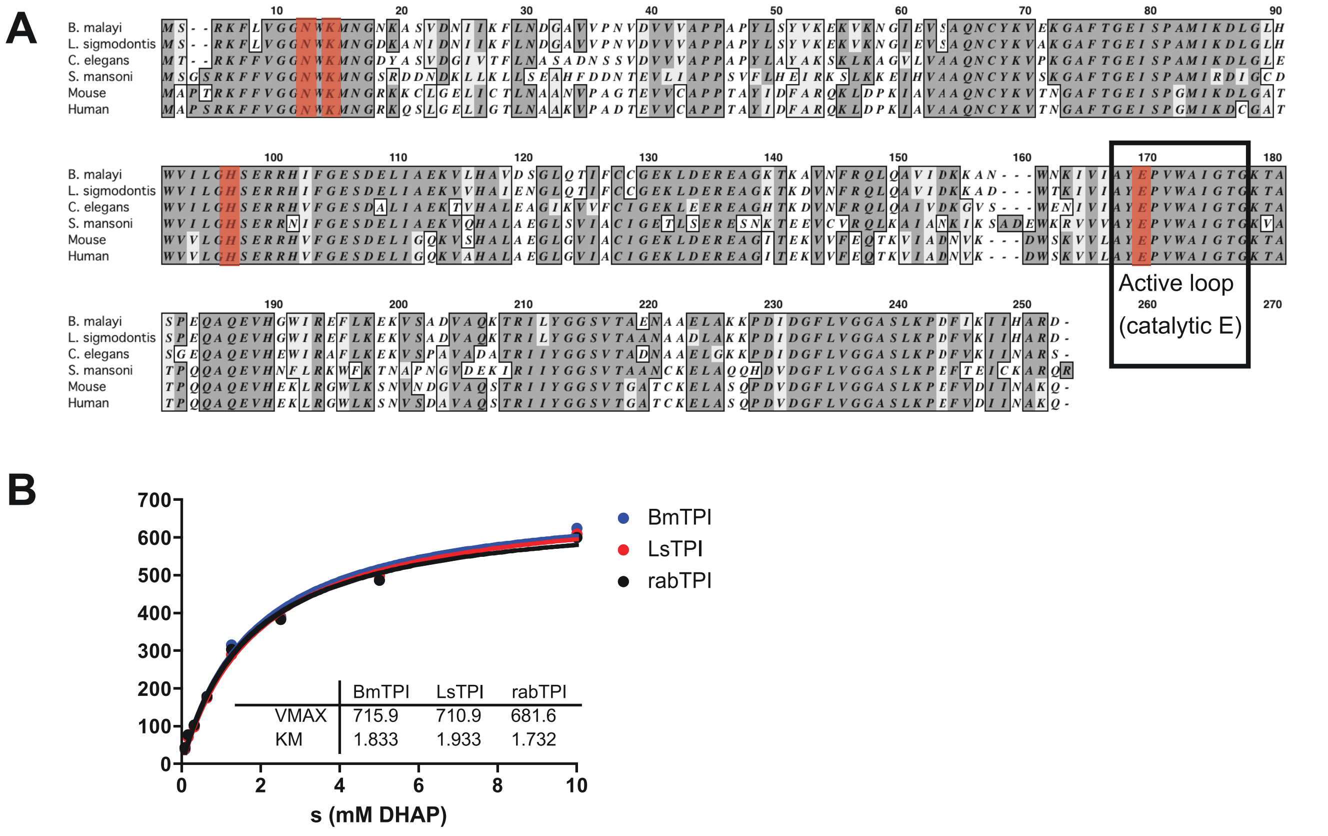

Figure 1. *Bm*-TPI is conserved in sequence and enzymatic function. A. Amino acid alignment of *Bm*-TPI (XP_001897269) with *Ls*-TPI (Hx2000032586), *Ce*-TPI (NP_496563), *Sm*-TPI (P48501), mouse TPI (NP_033441) and human TPI (NP_000356). **B**. Michaelis-Menten kinetics comparing activity of recombinant *Bm*- and *Ls*-TPI with native rabbit TPI. Results are representative of multiple batches of enzymes.
doi:10.1371/journal.ppat.1003930.g001

Antibody recognition of *Bm*-TPI by infected subjects

An important question is whether the prominent expression of *Bm*-TPI results in a strong antibody response in infected patients. We analysed serum samples from a cohort of *B. malayi*-exposed residents of Rengat, Sumatra, Indonesia that were classified into presumed uninfected subjects ("endemic normals"), asymptomatic microfilarial carriers, and patients with chronic filarial pathology who are generally amicrofilaraemic [35,36]. Individuals within each exposed group were found with positive IgG responses against *Bm*-TPI compared to sera from unexposed UK residents (**Fig. 3 A**). However, a greater proportion of infected individuals suffering from lymphatic pathology were seropositive (76%) compared to asymptomatic microfilaremics (48%) and endemic normals (42%), and the majority of strong responders were within the filarial pathology group. In contrast, no antibody reactivity was detected against mammalian TPI (using rabbit TPI which has 245/249 amino acid identity with the human protein) (**Fig. 3 B**). An isotype analysis of anti-*Bm*-TPI antibodies showed that reactivity was confined to the IgG1 and IgG4 isotypes (**Fig. 3 C–F**); notably IgG4 levels were higher to *Bm*-TPI in the pathology group, although the Mf+ individuals display far higher IgG4 levels to total *B. malayi* somatic antigens [35]. As we had previously detected little anti-*Bm*-TPI antibody reactivity using 2-D Western blots [6], the high level of reactivity found by ELISA indicated that the epitopes are predominantly conformational and denatured by SDS-PAGE electrophoresis, a conclusion supported by the lack of immunoreactivity in the vast majority of individuals to heat-treated *Bm*-TPI (**Fig. S1 A–D**).

Vaccination of rodents with filarial TPI does not confer protection against challenge infection

We next assessed whether vaccination of Mongolian jirds (*Meriones unguiculatus*), which are fully permissive to *B. malayi* infection [37], with *Bm*-TPI would generate protective immunity against challenge infection. In an initial experiment, animals were vaccinated three times with either *Bm*-TPI or control protein (BSA) in alum. Animals were infected intraperitoneally, with serum antibody titres and worm burdens being determined at 8 weeks post-challenge. All infected animals made strong IgG1 responses against a somatic extract of *B. malayi* adults (data not shown), but prior immunization with *Bm*-TPI induced >10 times higher IgG1 titres against this protein (**Fig. 4 A**). Despite this potent antibody response, and as shown in **Fig. 4 B**, there was no significant reduction in worm burdens at 8 weeks of infection in the *Bm*-TPI-immunized jirds (44±6.5, *vs* BSA, 55±7.0, p = 0.290). In a further experiment, we reasoned that a longer duration of infection might be required to see any protective effects induced by vaccination with a largely adult-specific secretory product. As such, jirds were immunised with *Bm*-TPI or BSA as before, challenged and assessed 21 weeks later. Vaccination again induced high titers of anti-TPI IgG1 (**Fig. 4 C**), but failed to provide any

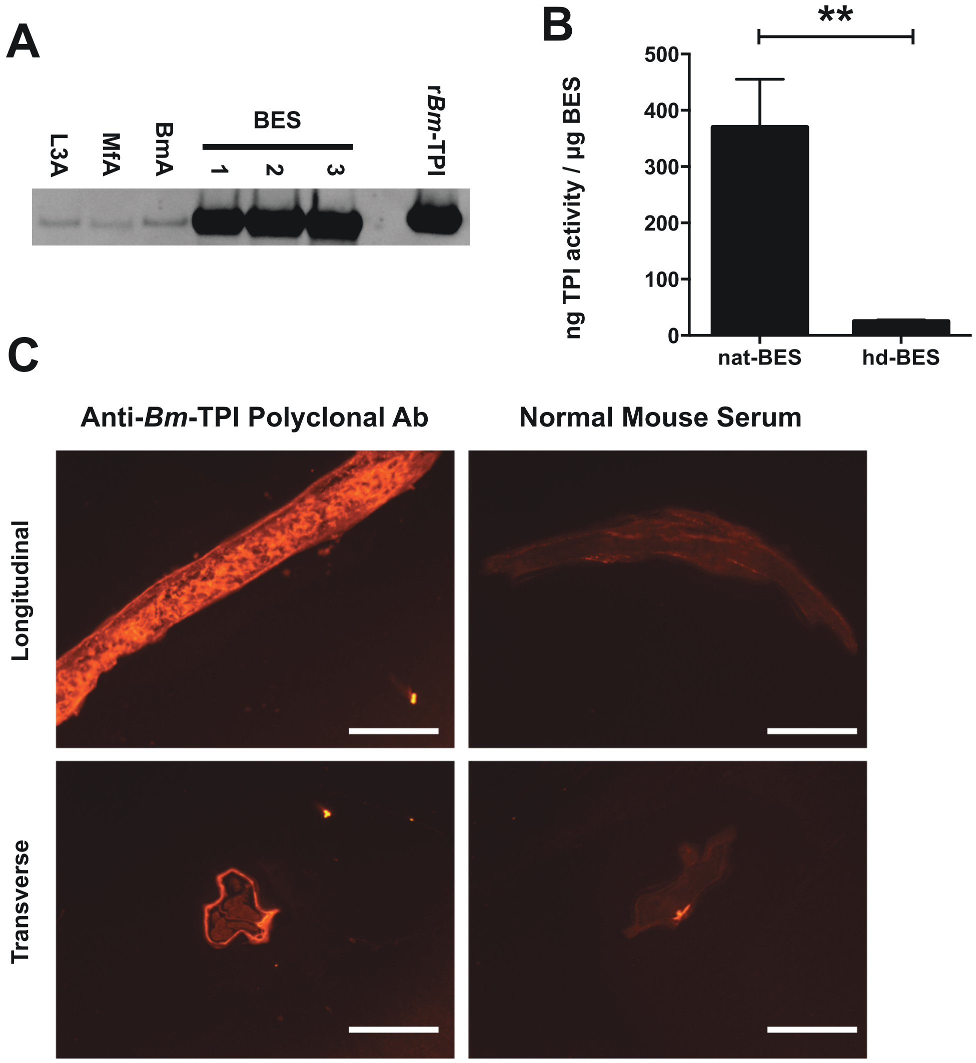

Figure 2. Adult worms preferentially secrete enzymatically active *Bm*-TPI. A. Western blot for *Bm*-TPI of 1 µg parasite extract (somatic extacts of L3 (L3A), Mf (MfA) and adult (BmA) or three independent batches of adult BES. Recombinant *Bm*-TPI included as a positive control. **B**. TPI activity in multiple independent batches of native BES or heat-denatured (hd)–BES. ** $p<0.01$ by *t*-test. **C**. Immunofluorescence of adult *B. malayi* female with polyclonal mouse anti-*Bm*-TPI (left panels) and control normal mouse serum (right panels) applied to longitudinal (Upper panels) and transverse (lower panels) sections. Scale bar represents 100 µm.
doi:10.1371/journal.ppat.1003930.g002

protection, and indeed both adult worm (**Fig. 4 D**) and peritoneal microfilariae numbers (**Fig. 4 E**) were slightly elevated compared to the BSA control.

The jird model is one that tests immunity of a rodent host to a human parasite, with parasites resident in the peritoneal cavity rather than their physiological niche of lymphatic vessels (for adult worms) and blood (for microfilariae). We therefore conducted a parallel test of protective capacity of TPI in a natural murine model of filarial infection, *L. sigmodontis*, which resides in the pleural cavity [34]. Mice were vaccinated three times with *Ls*-TPI in alum, and then challenged with *L. sigmodontis* L3. The results were similar to those observed with *B. malayi* in the jird: while specific anti-TPI antibodies were strongly boosted (**Fig. 4 F**), adult worm numbers were unchanged at day 70 post-challenge (**Fig. 4 G**) and when worm lengths were measured no differences were seen (data not shown). Moreover, circulating microfilarial numbers were not significantly diminished in immunized animals (**Fig. 4 H**).

Blockade of *Bm*-TPI does not impair parasite survival *in vitro*

One explanation for the poor level of protection induced by vaccination with *Bm*-TPI is the failure to generate high titres of neutralizing antibodies. Indeed, the sera from vaccinated jirds with high anti-*Bm*-TPI titres (**Fig. 4 B**) had limited ability to block *Bm*-TPI catalytic activity, leaving 75% of isomerase activity intact (**Fig. 5 A**). Similarly, polyclonal mouse serum raised to *Bm*-TPI effected only a slight reduction in enzyme activity (**Fig. 5 B**), whilst human sera from individuals strongly reactive to *Bm*-TPI (**Fig. 3**) failed to inhibit the enzyme at all (**Fig. 5 C**). This suggested that both immunisation and natural infection generated only limited amounts of anti-*Bm*-TPI antibodies directed at the active site. To confirm this, a panel of mouse monoclonal antibodies specific for *Bm*-TPI were generated. Only 2 of 23 (9%) anti-*Bm*-TPI clones were capable of blocking enzyme activity (**Fig. 5 D**), of which one anti-*Bm*-TPI mAb (clone 1.11.1, IgG1 isotype) was used in subsequent experiments.

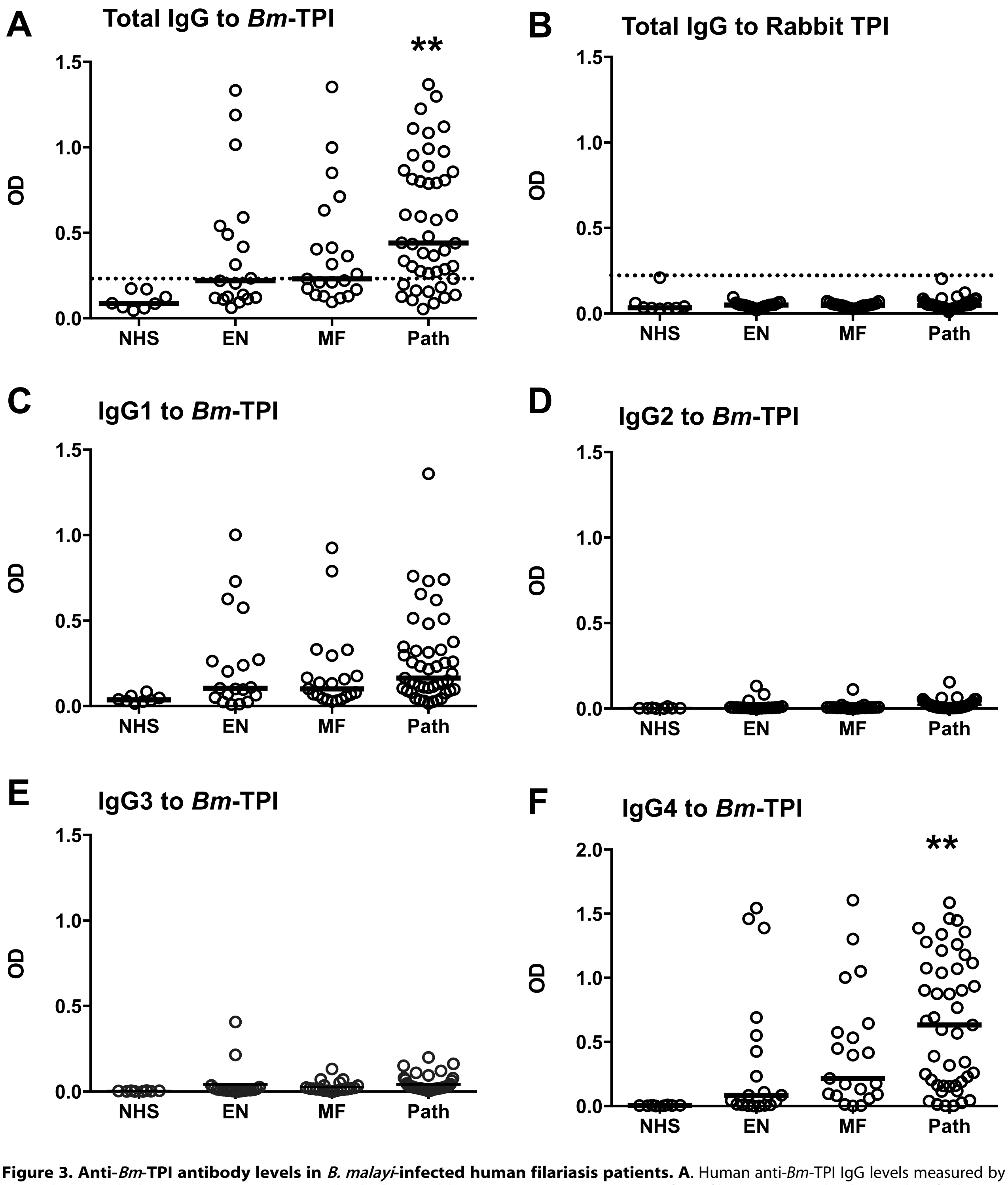

Figure 3. Anti-*Bm*-TPI antibody levels in *B. malayi*-infected human filariasis patients. A. Human anti-*Bm*-TPI IgG levels measured by ELISA (OD values). Each data point represents an individual normal human serum (NHS) or serum from filariasis-exposed subjects classified as endemic normal (EN), microfilaraemic (MF) or chronic pathology (Path). **B**. IgG ELISA responses of the same patient groups to mammalian (rabbit) TPI. **C–F**. IgG1-IgG4 isotype-specific anti-*Bm*-TPI levels in the same patient groups. ** $p<0.01$, significance compared to NHS by ANOVA. Lines in (A–F) represent median values.
doi:10.1371/journal.ppat.1003930.g003

MAb 1.11.1 was able to effectively block recombinant *Bm*-TPI enzymatic activity in a dose-dependent manner (**Fig. 5 E**), with a calculated Ki of <1 μg/ml for 100 ng *Bm*-TPI. In contrast, no blockade of either mammalian (**Fig. 5 E**), or perhaps surprisingly, *L. sigmodontis* TPI (**Fig. 5 F**) was seen. Most importantly, anti-*Bm*-TPI blocked native *Bm*-TPI as assessed by its ability to inhibit TPI activity present in BES (**Fig. 5 G**).

We then tested whether antibody inhibition of TPI enzymatic activity could cause parasite death *in vitro*. However, adult male and female *B. malayi* were able to survive in culture for sustained periods (≥3 days)

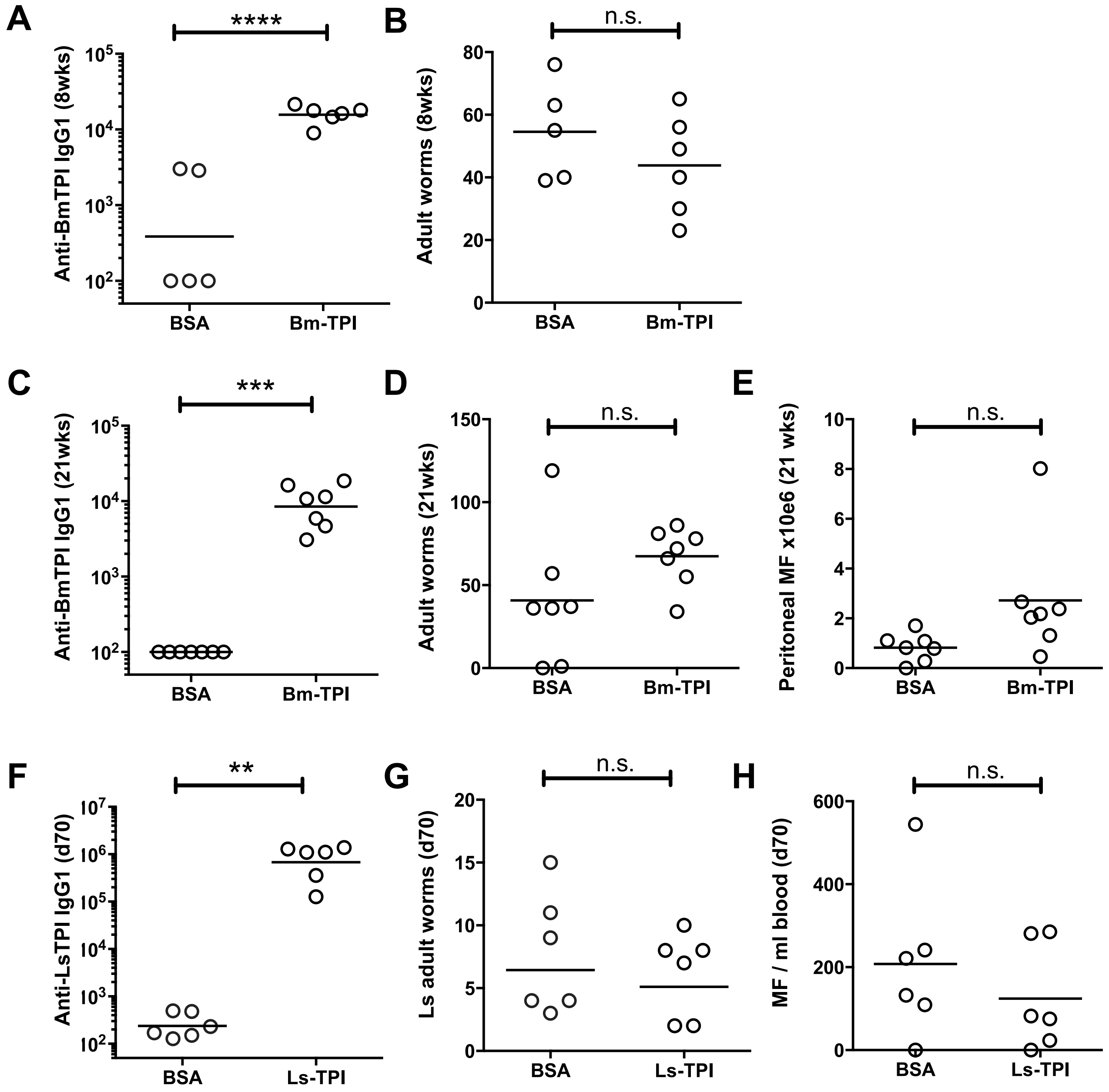

Figure 4. Vaccination with *Bm*-TPI does not curtail infection. **A**. Immunisation induces high titers of week 8 post-challenge anti-*Bm*-TPI IgG1 antibodies in vaccinated jirds, compared to animals immunised with BSA control. **B**. Week 8 post-challenge adult *B. malayi* worm burdens in *Bm-T*PI and BSA vaccinated jirds. **C**. Anti-*Bm*-TPI IgG1 titers remain high by week 21 post-challenge in vaccinated jirds, compared to BSA control animals. **D**. Week 21 post-challenge adult *B. malayi* worm burdens in *Bm-T*PI and BSA vaccinated jirds. **E**. Peritoneal *B. malayi* microfilarial counts in jirds at week 21 post-infection previously vaccinated with BSA or *Bm*-TPI. **F**. Immunisation induces high titers of day 70 post-challenge anti-*Ls*-TPI IgG1 antibodies in vaccinated BALB/c mice, compared to animals immunised with BSA control. **G**. Day 70 post-challenge adult *L. sigmodontis* worm burdens in *Ls-T*PI and BSA vaccinated BALB/c mice. **H**. Day 70 post-challenge blood *L. sigmodontis* microfilarial counts in *Ls-T*PI and BSA vaccinated BALB/c mice. Dotted lines in A, C and F represent background antibody titers naïve jird or mouse sera. n.s. non-significant, ** $p>0.01$, *** $p>0.001$, **** $p>0.0001$, by *t*-test.
doi:10.1371/journal.ppat.1003930.g004

in the presence of up to 500 μg/ml mAb clone 1.11.1 (data not shown). This suggested that whilst the antibody can inhibit secreted TPI, it cannot act directly on the parasite, and that *in vitro* worm survival over this period does not depend on TPI activity in the culture medium.

In vivo neutralization of *Bm*-TPI reduces microfilaraemia

Next, we tested whether MAb 1.11.1, with specific neutralizing ability, would alter the course of filarial infection *in vivo*. Mice were implanted intraperitoneally with 10 *B. malayi* adults (8 female, 2 male) and treated every 1–2 days with 200 μg anti-*Bm*-TPI mAb or IgG1 isotype control. Transfer of mAb 1.11.1 mAb established serum anti-*Bm*-TPI titres 35-fold greater than develop normally in response to infection, as seen in mice given the isotype control antibody (**Fig. 6 A**). Furthermore, transfer of 1.11.1 mAb conferred on recipient serum the ability to effectively block TPI enzymatic activity in vitro (**Fig. 6 B**). Despite this, live adult

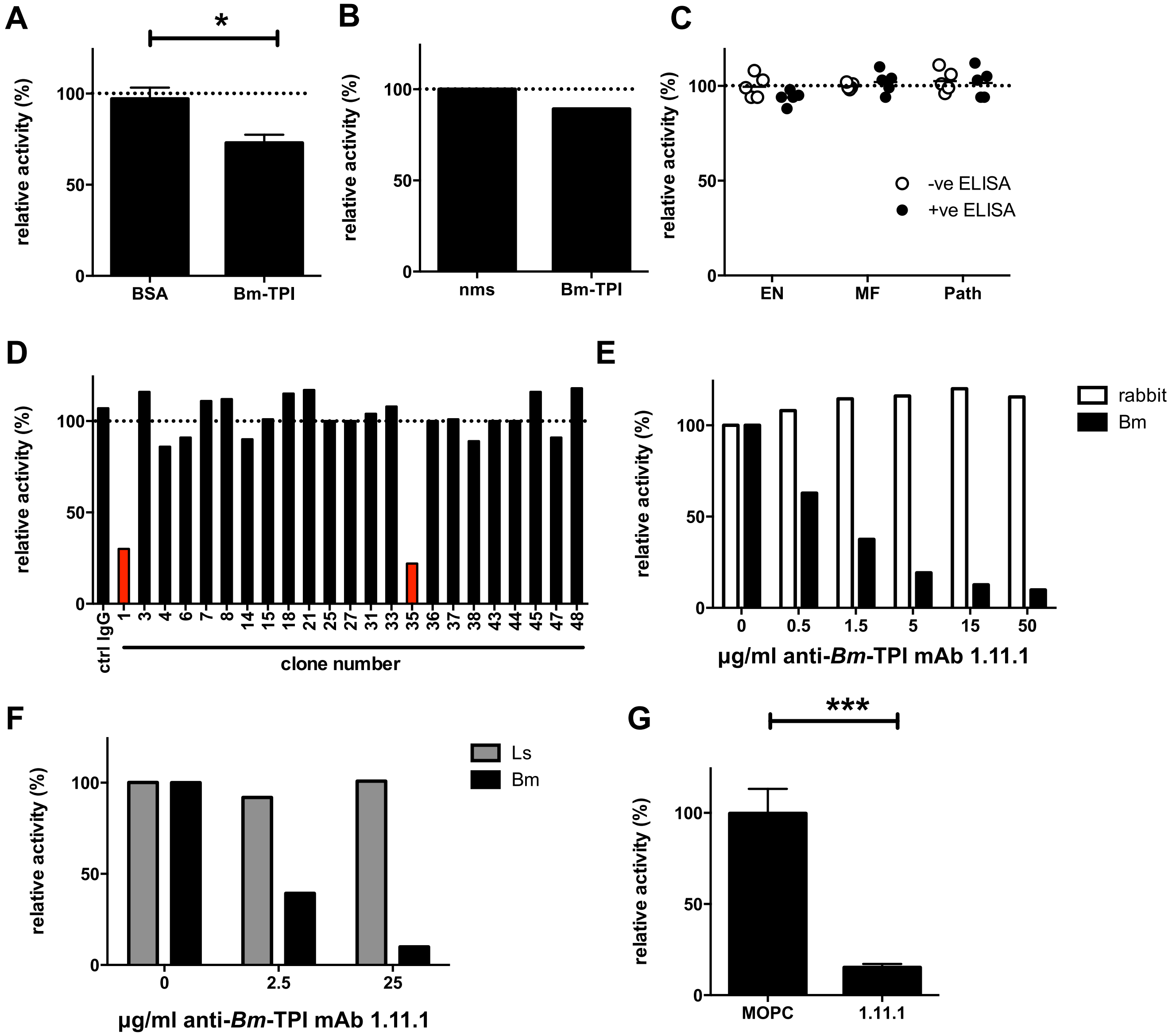

Figure 5. Generation of neutralizing antibodies to *Bm*-TPI. A. TPI enzyme activity in presence of 10% serum from jirds infected for 21 weeks with *B. malayi* following immunisation with BSA or *Bm*-TPI. * $p<0.05$ by *t*-test. **B**. TPI enzyme activity in presence of 10% polyclonal anti-*Bm*-TPI serum from BALB/c mice or naive mouse serum (nms). **C**. TPI enzyme activity in presence of 10% serum from human filariasis patients. Serum was used from the 5 strongest anti-*Bm*-TPI reactors (black circles) or 5 non-reactors (white circles) from each group (Fig. 3). **D**. Generation of antibody specificities that neutralise *Bm*-TPI activity is a relatively rare event. Ability of a panel of murine mAb specific for *Bm*-TPI (data not shown) to inhibit enzyme activity was determined. Clones with neutralising capacity are shown in red. **E**. Specificity of neutralizing mAb 1.11.1 for *Bm*-TPI (black bars) and not rabbit TPI (white bars). **F**. Specificity of neutralizing mAb 1.11.1 for *Bm*-TPI (black bars) and not *Ls*-TPI (grey bars). A, B, E and F are representative of multiple batches of recombinant enzyme. **G**. Neutralisation of native *Bm*-TPI activity in BES (4 independent batches) by mAb 1.11.1. MOPC31C IgG1 myeloma protein was used as a control. Dotted lines in (A–D) represent enzyme activity in the absence of serum or antibody (normalised to 100%).*** $p<0.001$ by *t*-test.
doi:10.1371/journal.ppat.1003930.g005

worms were recovered from the peritoneal cavities of both groups of infected mice after 28 days (**Fig. 6 C**) with no significant differences in male or female numbers (data not shown). In contrast, numbers of peritoneal microfilariae (Mf) were significantly reduced in anti-*Bm*-TPI-treated mice, being 69.5% lower than in animals given isotype control (**Fig. 6 D**) indicating that *Bm*-TPI activity is required for either the release or survival of live Mf.

Using Mf obtained from non-immunised jirds, we next demonstrated that *in vitro Bm*-TPI blockade was unable to kill Mf (**Fig. 6 E**), indicating that the antibody is not directly toxic to Mf, and that neutralisation of TPI is not detrimental to this stage of the parasite. Likewise, *in vitro Bm*-TPI blockade did not reduce Mf production by adult females obtained from non-immunised jirds (**Fig. 6 F**). Instead, when we cultured adult females from the peritoneal cavity of mice following *in vivo* anti-*Bm*-TPI or isotype treatment, parasites from anti-*Bm*-TPI treated mice produced ~60% fewer MF *in vitro*, consistent with *Bm*-TPI blockade compromising parasite fitness in terms of female reproductive output *in vivo* (**Fig. 6 G**). In particular, a much greater proportion

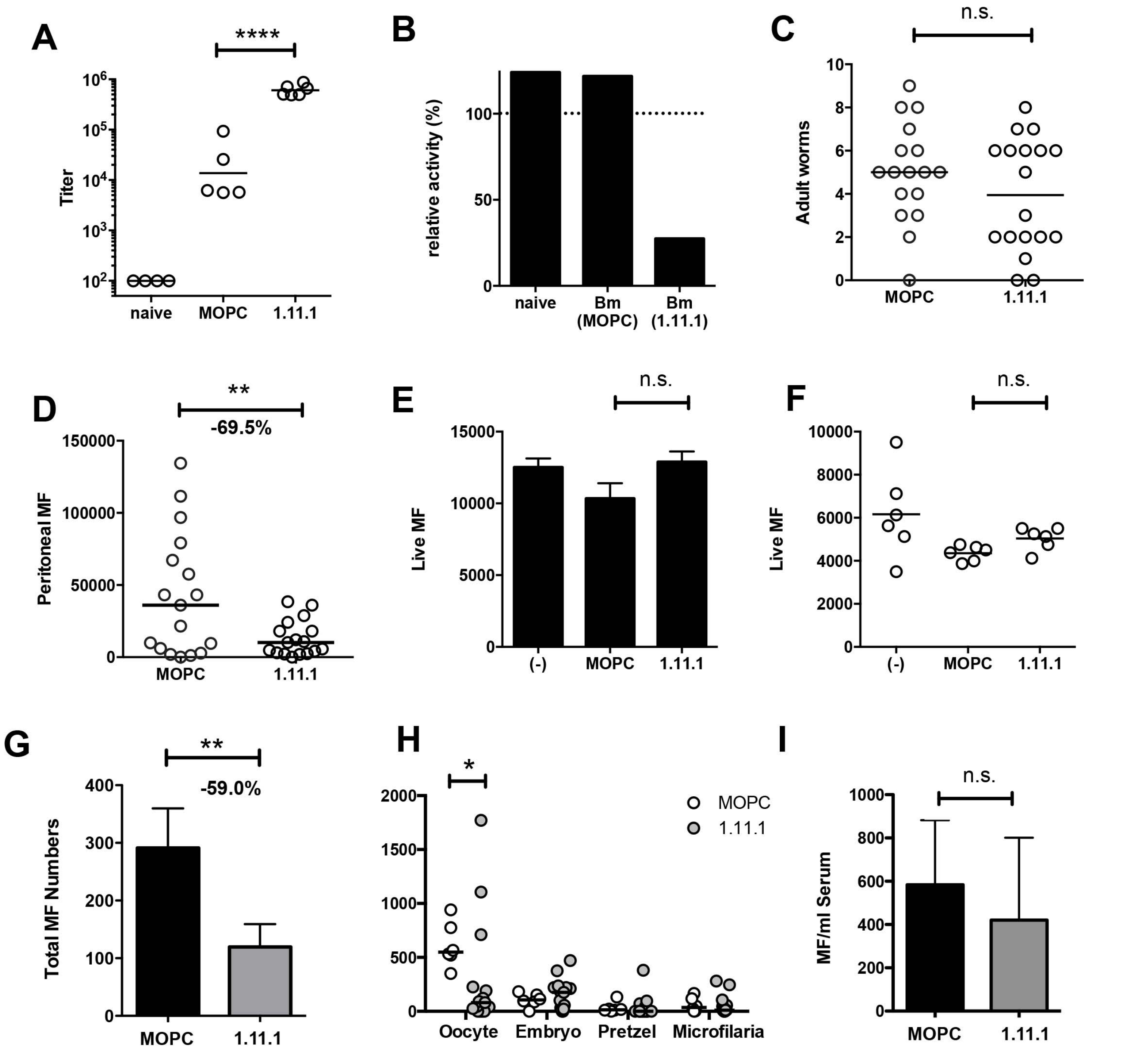

Figure 6. *In vivo Bm*-TPI blockade reduced microfilarial production by adult females. A. Serum titers of anti-*Bm*-TPI antibodies following multiple injections (16×) with 1.11.1 anti-*Bm*-TPI mAb or control IgG1 myeloma protein MOPC31C in mice 28 days following transplant of adult *B. malayi* worms. Representative of two experiments. **** $p<0.0001$ by ANOVA. **B**. Ability of serum from recipient mice to neutralize *Bm*-TPI activity. Sera were compared from naïve mice or day 28 post-Bm adult implant mice treated with MOPC or 1.11.1 anti-Bm-TPI mAb as (A). Dotted line indicates enzyme activity in absence of serum. **C**. Day 28 peritoneal worm burdens in recipients of MOPC31C or 1.11.1 anti-*Bm*-TPI Mab as (A). **D**. Day 28 peritoneal microfilarial counts in recipients of MOPC31C or 1.11.1 anti-*Bm*-TPI Mab as (A). Data in C and D are combined results from 3 independent experiments, with 5–6 mice per group. ** $p<0.01$ by *t*-test. **E**. Live MF numbers following 3 day *in vitro* culture alone, with MOPC31C or with 1.11.1. Initial MF input was 15,000 and data is from 3–4 wells per treatment, and is representative of two experiments. **F**. Live MF numbers recovered from individual female worms obtained from the peritoneal cavity of untreated jirds. Worms were cultured for 2 days alone, with MOPC31C or with 1.11.1, and is representative of two experiments. **G**. Live Mf numbers by individual female worms (2 day cultured) obtained from the peritoneal cavity of mice injected multiple times with MOPC31C or with 1.11.1. for 14 days. * $p<0.05$ by Mann-Whitney. Data are pooled from two independent experiments. **H**. Embryogram of uterine contents of individual adult female *B. malayi* parasites recovered from peritoneal cavity of mice (n = 4) injected multiple times for 14 days with MOPC31C or 1.11.1 anti-TPI antibody. **I**. Microfilarial numbers in blood 24 hours following i.v. transfer into mice receiving control MOPC31C and 1.11.1 anti-TPI antibodies.
doi:10.1371/journal.ppat.1003930.g006

of adult female worms from anti-TPI-treated jirds completely failed to release live Mf during *in vitro* culture (45.2% compared to 12.5% in isotype-treated controls).

Analysis of uterine contents from individual female *B. malayi* revealed that TPI blockade led to a significant decrease in the number of unfertilised oocytes (**Fig. 6 H**). Whilst oocytes were the predominant developmental stage in females obtained from isotype treated mice (**Fig. S2 A**), anti-Bm-TPI treatment led to the accumulation of smaller developmental stages that appeared damaged or partially degraded (**Fig. S2 B**). In contrast, no significant difference

was observed in the number of Mf transferred directly into antibody-treated mice (**Fig. 6 I**) again implying that TPI blockade targets female egg production, thereby reducing subsequent release of live Mf.

Macrophage expansion in TPI-neutralised mice. To establish if the stronger immunity in the TPI-neutralised setting is associated with changes in immune cell types, we studied the frequency and phenotype of key cell populations in *B. malayi*-implanted mice. Administration of 1.11.1 did not affect overall cell recruitment to the peritoneal cavity infection site (**Fig. 7 A**), but TPI blockade resulted in a greater proportion of $CD11b^{+}F4/80^{+}$ macrophages within this compartment (**Fig. 7 B**). Analysis of markers of alternative activation of macrophages showed that Chi3L3 (Ym1) and RELMα expression was uniformly high in $CD11b^{+}$ cells from all infected mice, irrespective of treatment (**Fig. 7 C**), indicating alterations in quantitative expansion rather than the qualitative nature of the macrophage population. In contrast to increased frequencies of macrophages, we also found reduced eosinophilia in the anti-TPI-treated mice (**Fig. 7 D**).

Within the $CD4^{+}$ T cell subset, Th2 responses measured by total IL-4-expressing $CD4^{+}$ T cells, also showed little difference in antibody recipients (**Fig. 7 E**), but there was a significant reduction in IFN-γ expression, indicating diminished Th1 responsiveness in the absence of TPI enzymatic activity (**Fig. 7 F**). To test whether Bm-TPI directly stimulates the expansion of Th1 cells, transgenic DO11.10 ovalbumin-specific T cells were co-cultured with ovalbumin peptide and TPI, or co-transferred to naïve mice together with ovalbumin antigen and TPI i.p. However, cytokine responses in TPI recipients were unaltered both in vitro and in vivo (**Fig. S3**) arguing that the effects of TPI on the effector T cell compartment occur in the setting of an active parasite infection. Finally, we examined the activation of Foxp3-expressing regulatory T cells in mice receiving *B. malayi* implants which has been previously reported, including the induction of CD103 expression [38]. Frequencies of $Foxp3^{+}$ Tregs expressing the marker CD103 were elevated in all infected mice irrespective of 1.11.1 administration (**Fig. 7 G**).

Discussion

Parasite mediated-immunomodulation relies on products secreted into the environment of the pathogen *in vivo*, and the analysis of *in vitro* released "excretory-secretory" (ES) proteins has provided an approximation of the spectrum of released macromolecular components. We and others have identified *Bm*-TPI as a dominant product secreted by adult *B. malayi* worms [6–9], and here confirm not only the preferential secretion of this enzyme by live adult worms *in vitro*, but show, by its antigenicity in infected mice and humans, that it is exposed to the immune system *in vivo*. How TPI is secreted, in the absence of a signal peptide, remains unclear and its ubiquitous expression throughout the somatic tissues of *B. malayi* does not provide any pointers to a particular route of secretion. Possibly, as secretion is far higher in female worms than males [7], TPI could be released along with microfilariae from the female genital tract.

Filarial TPIs, from both *B. malayi* and *L. sigmodontis*, are active enzymes with catalytic properties very similar to those of mammalian homologues. Immunologically, however, TPIs from mammals and nematodes are non-cross-reactive, and indeed we generated monoclonal antibodies capable of distinguishing between the two filarial enzymes. Hence, in human infections anti-*Bm*-TPI antibodies were not found to be auto-reactive with self TPI, suggesting that the pathologies of lymphatic inflammation, edema and fever are not linked to an autoimmune reaction against human TPII. In this respect, filarial infection differs from that of *Trypanosoma cruzi*, which induces auto-antibody production against host TPI [39].

When comparing the levels of anti-*Bm*-TPI between infected individuals we noted that titres are greater in cases of filarial pathology in whom circulating microfilariae are generally absent. Because of this, we tested the potential of *Bm*-TPI to provoke protective immunity against *B. malayi* in animal models. In the jird, *M. unguiculatus*, which is fully susceptible to infection with the mosquito-borne L3 stage, both total worm and Mf numbers were unchanged in vaccinated animals following peritoneal infection. However, vaccination did not generate high levels of neutralizing antibody in terms of the catalytic activity of the enzyme, which may be more efficient than removal of TPI by complexing and opsonization. By screening a large panel of murine monoclonals, we selected a neutralizing antibody that conferred, in a mouse peritoneal implantation model, immunity against the Mf stage.

We also noted that TPI blockade inhibits the development of eggs within the adult female worm, reflecting a loss of fitness in the parasite that may have either or both a metabolic or immunological cause. Several other regimes have been shown to limit the release of viable microfilariae by filarial parasites. In this regard, antibiotic-mediated depletion of endosymbiotic *Wolbachia* causes degeneration of *B. malayi* oocytes, embryos and microfilariae [40,41], although in this setting parasite metabolism is likely to be more heavily compromised. An immunological cause of reduced parasite fecundity can also be illustrated in mice immunised with *L. sigmodontis* Mf, >70% of which fail to develop microfilaraemia, due to an inhibition of embryogenesis to the pretzel stage [42].

The selective effect on microfilarial levels recapitulates a consistent, but unexplained, feature of filarial nematode infections. In both humans and animal models, cryptic amicrofilaraemic infections can occur in which immunity appears to operate only against the microfilarial stage. For example, cats infected with *B. pahangi* often became Mf-negative and yet remained seropositive for circulating filarial antigen and were found to harbour live adult worms at autopsy [43]. Similarly, circulating antigen tests in humans identify a significant proportion of Mf-negative infected subjects. However, in the majority of cases, it appears that natural infection does not generate blocking antibody to TPI suggesting the possibility that the active site has in some manner evolved to minimise stimulation of neutralising antibody. The surprising finding that such antibodies do not cross-react between the highly conserved TPIs from two related filarial species reflects a further unusual property of these proteins.

To establish whether TPI enzyme activity is essential for its extracellular function, and whether only blocking antibodies can be protective, it would be desirable to test multiple panels of monoclonals and to conduct experiments with pharmacological inhibitors of filarial TPI. Future studies may also develop blocking antibodies against *L. sigmodontis* TPI which would permit the analysis of the biological role of TPI during the full course of infection in mice, as the murine antibodies we describe here cannot be administered to other rodent species for extended periods of time.

In addition, species-specific chemical TPI inhibitors have been described which target non-conserved amino acids, particularly at the dimer interface e.g. *Trypanosoma cruzi* [44], *Trypanosoma brucei* [45], *Plasmodium falciparum* [46] and *Giardia lamblia* [47]. Small molecule inhibitors may be superior to antibody-mediated blocking as they offer the advantage of penetration into the parasite itself, rather than just inhibition of secreted form, and are likely to be more effective against the adult worms which, as we show, survive even in the face of very high neutralising Ab titres.

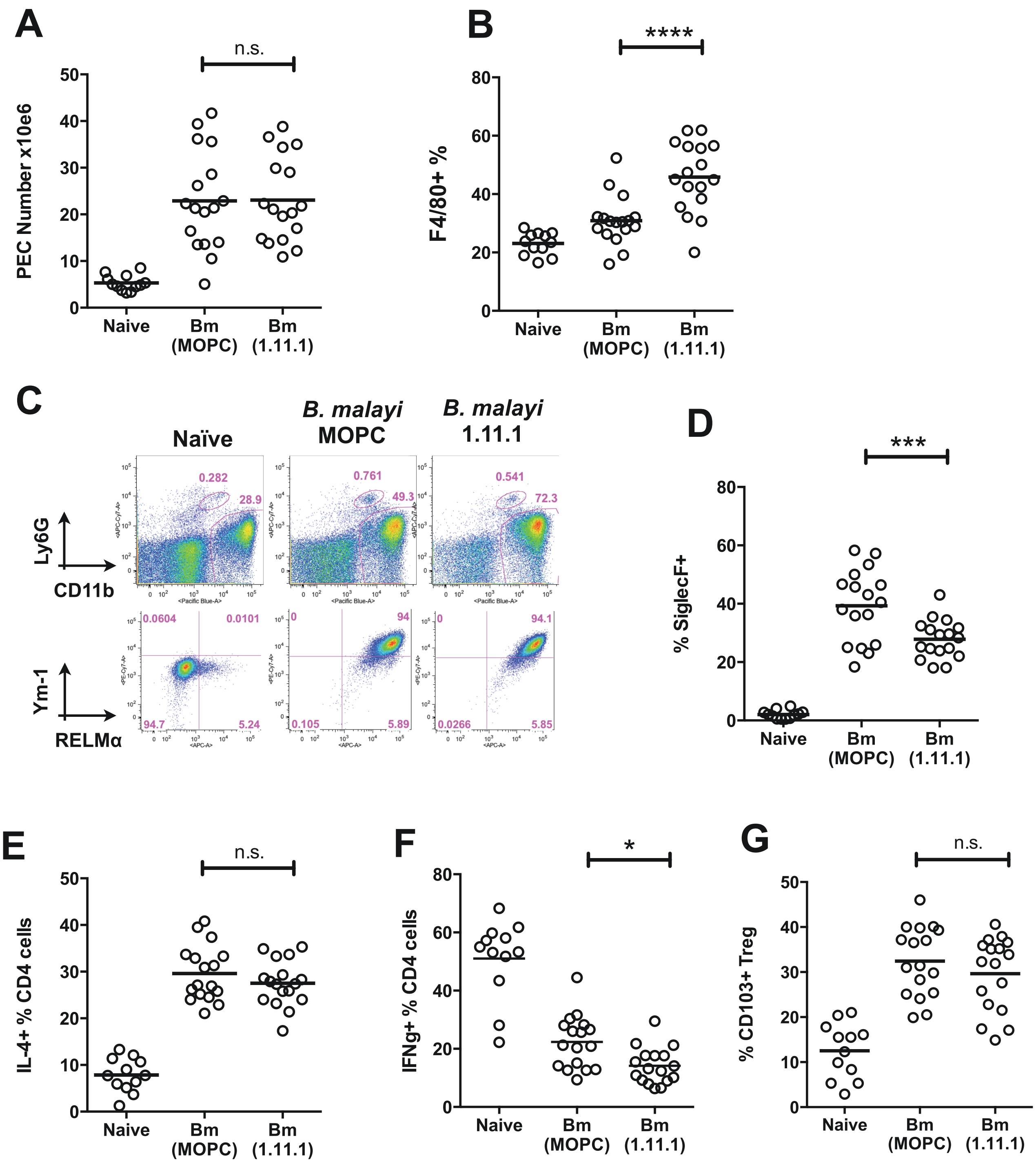

Figure 7. Altered T cell, eosinophil and macrophage responses in mice receiving neutralising anti-Bm-TPI monoclonal antibody. A. Peritoneal cell recruitment in recipients of 1.11.1 anti-*Bm*-TPI monoclonal antibody or control IgG1 myeloma protein MOPC31C, in mice 28 days following transplant of adult *B. malayi* worms. **B**. Peritoneal CD11b$^+$ F4/80$^+$ macrophages as in (A). **C**. Intracellular expression of Ym-1 and RELMa by peritoneal macrophages (CD11b$^+$ F4/80$^+$ Ly6G$^-$ siglecF$^-$) in mice 14 days following transplant of adult *B. malayi* parasites with 1.11.1 anti-*Bm*-TPI monoclonal antibody or control IgG1 myeloma protein MOPC31C. Representative of 4 mice per group. **D**. Peritoneal CD11b$^+$ siglecF$^+$ cells as in (A). **E**. Intracellular IL-4 production by CD4$^+$ peritoneal T cells as in (A) **F**. Intracellular IFN-γ production by CD4$^+$ peritoneal T cells as in (A). **G**. Frequency of CD103$^+$ expression among Foxp3$^+$ Tregs as in (A). Data in (A–B, D–G) are pooled from 4 independent experiments.
doi:10.1371/journal.ppat.1003930.g007

The *in vivo* consequences of TPI neutralisation may link poor microfilarial survival with shifts in the anti-parasite immune response. Thus, the reduction in IFN-γ production by peritoneal cavity CD4$^+$ T cells may be an indirect effect of diminished numbers of microfilariae, as this stage (unusually) induces Th1 responsiveness [48–50]. Similarly, the reduced eosinophilia could

reflect weaker stimulation from the microfilarial stage. Interestingly, this argument would suggest that microfilariae normally dampen macrophage expansion in vivo, a point which has yet to be experimentally investigated.

The finding that TPI is also secreted by plant-parasitic nematodes [51], and the importance of glycolysis in adult *B. malayi* [31], are consistent with TPI release facilitating adult worm metabolism and being required for optimal fecundity and Mf production *in vivo*. It is also possible that the heightened frequency of alternatively-activated macrophages imposes immunological damage on adult worms, thereby reducing their ability to reproduce, and such damage may of course be more easily achieved if the target is also metabolically compromised. In any event, our data present a remarkable immunological strategy for transmission in the filarial nematode: adult females release TPI which promotes oogenesis, and so increases the number of their offspring. Hence, in the mouse model at least, TPI neutralisation inhibits this process and reduces the microfilarial burden.

Materials and Methods

Animals, human samples, parasites and production of BES

BALB/c mice and *Meriones unguiculatus* jirds were bred in-house. *B. malayi* (obtained originally from TRS Laboratories, Athens, Georgia, USA) was maintained in *Aedes aegypti* mosquitos and jirds. Infective larvae (L3) were recovered from mosquitos 12–14 days following feeding on microfiariae- (Mf-) containing blood, as detailed previously [52]. Jirds were infected with up to 600 L3; adult worms and microfilariae were recovered from the peritoneum approximately 4 months later. The *Litomosoides sigmodontis* life cycle was maintained, and L3 larvae or adult worms recovered, as described previously [53]. Somatic extracts of worms (from adults, BmA; from L3, L3A and from Mf, MfA) and BES were produced as previously described [6].

Ethics statement

All animal protocols adhered to the guidelines of the UK Home Office, complied with the Animals (Scientific Procedures) Act 1986, were approved by the University of Edinburgh Ethical Review Committee, and were performed under the authority of the UK Home Office Project Licence number 60/4105. Human serum samples were taken from archived stocks derived from a study in Rengat, Indonesia that has been previously described [54] and in which informed consent was obtained from all patients before clinical and parasitologic investigation and blood withdrawal in accordance with the guidelines of the Indonesian Department of Health and Human Services.

Cloning and recombinant expression of *Bm*-TPI and *Ls*-TPI

Total RNA was extracted from adult mixed sex *B. malayi* and *L. sigmodontis* using TRIzol (Invitrogen), and reverse transcribed with MMLV reverse transcriptase (Stratagene) using standard protocols. A partial sequence (nt 1–664) for Ls-TPI (LS00587) was obtained from NEMBASE v4 (http://www.nematodes.org/nembase4/index.shtml). The missing 3′ end was obtained by 3′ RACE using Invitrogen Gene Racer Core kit with RACE-ready cDNA and the forward primer ATG TCT CGA AAG TTT CTA GTT as previously described [55]. The resultant full-length nucleotide sequence has been submitted to the European Nucleotide Archive with the Accession Number HG329626. The following PCR primers were used for amplification; *Bm*-TPI forward primer CAT ATG TCG CGA AAA TTT CTT, *Ls*-TPI forward primer CAT ATG TCT CGA AAG TTT CTA GTT, *Bm*-TPI and *Ls*-TPI reverse primer CTC GAG ATC ACG TGC ATG AAT AAT TT (restriction sites are underlined). PCR conditions were as follows: 35 cycles of 95°C 30 sec, 60°C 30 sec, and 72°C 2 min. Reaction products were separated on 1% agarose gels, visualised using ethidium bromide, and the 750-bp amplicons excised and purified (QIAquick gel extraction, Qiagen). PCR products were cloned into pGEM-T vector (Promega) and transformed into *E. coli* JM109 (Promega) for overnight colony formation. Minipreps from positive colonies were sequenced. An internal *Nde1* site was removed in both *Bm*-TPI and Ls-TPI by PCR-based site-directed mutagenesis (CATATG replaced with CATACG, a synonymous mutation, in-frame codon underlined), using 50 ng of parental plasmid, Deep Vent DNA polymerase (New England Biolabs) and the following PCR primers : *Bm*-TPI forward primer CCT TAT TTA TCA TAC GTT AAG GAG AAA, *Bm*-TPI reverse primer TTT CTC CTT AAC GTA TGA TAA ATA AGG, Ls-TPI forward primer CCA TAT TTG TCA TAC GTC AAG GAA AAA GTT, Ls-TPI reverse primer AAC TTT TTC CTT GAC GTA TGA CAA ATA TGG. PCR conditions were as follows: 18 cycles of 95°C 30 sec, 55°C 1 min, and 75°C 8 min. Reaction products were digested with *Dpn1* (New England Biolabs) for 2 hours at 37°C to remove parental plasmid, purified as for plasmid preps, and then used to transform *E. coli* JM109, and grown as before. Coding sequences for *Bm*-TPI and *Ls*-TPI were ligated into linearised pET29c (Novagen) following digestion with *Nde1* and *Xho1*. Protein expression was induced in BL21(DE3) cells with 1 mM IPTG for 3 hours at 37°C. Bacteria were pelleted and lysed in Bug Buster supplemented with 25 U/ml benzonase (Novagen) for 20 min at room temperature. C-terminal His-tagged proteins were purified by metal affinity chromatography using Hi-Trap chelating Hp columns on an AKTAprime (GE Healthcare). Eluted fractions containing recombinant TPI protein were pooled and dialysed into PBS. Endotoxin was removed using Detoxi-Gel Endotoxin Removing Columns (Thermo Scientific). Recombinant TPI was stored at 2 mg/ml at −80°C.

Enzymatic assays

Enzymatic activity of TPI was determined in the reverse direction (conversion of DHAP to G3P) as described by Lambeir *et al* [56]. Standard reaction conditions were 100 mM TEA-HCl pH 7.6, 1 mM EDTA, 0.16–10 mM DHAP (1.5 mM if not indicated), 1 mM NAD^+, 5 mM disodium hydrogen arsenate and 10 µg rabbit GAPDH. Reactions were initiated with 100 ng TPI in a total volume of 150 µl. Rabbit TPI and all above reagents were from Sigma. The initial reaction rate was calculated by the change in NADH absorbance at 340 nm with a Nanodrop 2000 (Thermo Scientific). Enzyme activity was calculated in units (1 U = 1 µmol substrate formation min^{-1} μg^{-1} enzyme at 25°C). Calculations were confirmed using a NADH standard curve (Sigma). To determine the level of TPI activity in BES, reactions were initiated with 500 ng of BES or heat denatured BES (95°C 30 min), and compared to 25–400 ng of *Bm*-TPI. To assess the inhibitory potential of serum from immunised animals, enzymatic assays were performed in the presence of 10% test or control serum. Enzyme blockade by monoclonal antibodies was assessed in the range 0.5–50 µg/ml, and an isotype control mouse IgG1 MOPC31C was used where indicated. In these instances, enzymatic activity was calculated as percentage activity compared to the relevant control.

Human, jird and mouse antibody and cytokine ELISA

Recombinant *Bm*-TPI, rabbit TPI (Sigma), BSA or BmA were coated (1 µg/ml in 0.06 M carbonate buffer pH 9.6) onto

Maxisorb 96 well Immunoplates (Nunc) overnight at 4°C. Plates were blocked in 2% BSA (mouse and human sera) or 1% casein (jird sera) in tris-buffered saline/0.05% tween 20 (TBST) for 2 hours at 37°C. Human IgG responses to *Bm*-TPI were tested using sera (1/100 dilution) from a previously characterised Indonesian *B. malayi* endemic study population [57]. Individuals were classified into three clinical groups, elephantiasis (pathology) patients, asymptomatic microfilaraemics, and endemic normals. Normal human sera (NHS) were obtained from non-exposed UK residents. IgG binding was detected using peroxidase labelled anti-human IgG (1/1000, DakoCytomation), or anti-human IgG1, IgG2, IgG3 or IgG4 (1/5000, The Binding Site). Plates were developed using ABTS peroxidase substrate (KPL), and measured with an Emax microplate reader (Molecular Devices). Samples were considered positive if they exceeded the mean value plus 3 standard deviations of nonendemic human serum samples. Jird sera (1/100 dilution) antibody levels were measured using peroxidase labelled anti-mouse IgG1 (1/2000, Southern Biotech). BALB/c anti-*Bm*-TPI titres were determined with doubling dilutions of sera (1/50 onwards) and anti-mouse Ig peroxidase (1/2000, DakoCytomation).

Polyclonal and monoclonal antibody production

For polyclonal antibody production, BALB/c mice were immunised i.p. with 50 µg *Bm*-TPI in alum, and then boosted with 10 µg *Bm*-TPI i.p. in alum on days 28 and 35. Serum was recovered on day 42. For monoclonal antibody production, BALB/c mice were immunised with 50 µg *Bm*-TPI, and then boosted with 1 µg *Bm*-TPI in PBS i.v. on days 28, 29 and 30. Spleens were recovered on day 32 and fused with SP2 as before [58]. Cells were screened for *Bm*-TPI binding by ELISA, and positive wells were tested for *Bm*-TPI blocking ability in the above enzymatic assay. From this a blocking hybridoma was obtained, and cloned through two rounds of limiting dilution, resulting in anti-*Bm*-TPI clone 1.11.1. This was found to be an IgG1 using mouse antibody isotype kit (Isostrip, Roche). Monoclonal antibody was purified from culture supernatants using HiTrap protein G HP columns and an AKTAprime, and dialysed into PBS. Control mouse IgG1 MOPC31C was produced in the same way from cells sourced from ECACC.

Western blots

BES and somatic extracts of L3, MF and mixed adult *B. malayi* (1 µg) were run on SDS-PAGE gels and blotted onto nitrocellulose membranes as previously described [6]. Following blocking in 5% milk powder/TBST (2 hours room temperature), membranes were probed overnight at 4°C with 1/500 mouse polyclonal anti-*Bm*-TPI, washed extensively in TBST and the incubated with 1/2000 rabbit anti-mouse Ig HRP (1 h, room temperature; DakoCytomation). Following further washing in TBST, blots were developed using ChemiGlow West (Alpha Innotech) and imaged using a FluorChem SP (Alpha Innotech).

Immunohistochemistry

Adult *B. malayi* were mounted in Cryo-M-Bed (Bright Instruments), frozen on dry ice, and 5 µm sections cut using a Leica CM1510S cryotstat. Air dried sections were fixed in 100% acetone (10 min), washed twice with PBS (20 min), and stained in a humidified chamber with 1/100 dilution mouse anti-*Bm*-TPI sera (generated as above) in 1% FCS/PBS (1 hour at room temperature). Control sections were similarly treated with naïve mouse sera. Following extensive washing in PBS, sections were incubated with 1/100 goat anti-mouse IgG TRITC (Sigma) as above, washed in PBS, then mounted with Vectashield (Vector labs). Sections were analysed with an Olympus BX50 fluorescent microscope and Openlab software (PerkinElmer).

Vaccination regimes

Meriones unguiculatus jirds were immunized with 200 µg of *Bm*-TPI or BSA i.p. in alum adjuvant and then boosted with sub-cutaneous injections of 50 µg protein in alum at weeks 5 and 6. Jirds were challenged with 190 *B. malayi* L3 i.p. at week 8 post-immunisation. Infection was allowed to progress 8 weeks in the first experiment and 16 weeks in the second experiment. For *Ls*-TPI vaccination, BALB/c mice were immunised with 50 µg Ls-TPI or BSA in alum, and then boosted on weeks 4 and 5 with 25 µg protein in alum. Mice were infected sub-cutaneously on week 7 post-immunisation with 30 *L. sigmodontis* L3, and infections were terminated at week 10 post-infection.

In vivo Bm-TPI neutralisation and embryograms

Surgical implant of adult *B. malayi* (8 females and 2 males) into the peritoneal cavity of BALB/c mice was performed as previously described [50]. Mice were given 200 µg of anti-*Bm*-TPI clone 1.11.1 mAb or MOPC31C isotype control every 1–2 days over the course of a 14–28 day infection. Embryograms were performed on recovered female parasites exactly as [42]. For *in vivo* microfilariae transfer, Mf from the peritoneal cavity of infected jirds (1×10^5) were transferred i.v. in 200 µl RPMI1640 into BALB/c recipients, which were then injected with 200 µg antibody as above. Circulating Mf numbers were determined by tail bleed 24 hours later.

FACS analysis, cell culture and transfer

Cells were recovered from infected mice by peritoneal wash [38], washed into FACS buffer (PBS +0.5% BSA +0.05% sodium azide) Fc receptors blocked with 0.5 mg/ml rat IgG on ice for 10 minutes, then stained variously with anti-CD11b Pacific Blue (Biolegend; clone M1/70) anti-siglecF (PE or PE CF-594 conjugates, BD Pharmingen; clone E50-2440), anti-Ly6G APC-Cy7 (Biolegend; clone 1A8) and anti-F4/80 (FITC or PerCP conjugates, Biolegend; clone BM8). For macrophage alternate activation analysis, cells were fixed and permeablised (eBioscience, as per manufacturer's instructions) before intracellular staining with anti-RELMα (unlabeled rabbit polyclonal; PeproTech, followed by rabbit Ig labeling reagent; Invitrogen) and anti-Ym-1 (biotin-conjugated mouse chitinase 3-like 3; R&D, followed by streptavidin PE-Cy7; Biolegend). For intracellular cytokine staining, peritoneal cells were re-stimulated *ex vivo* in complete RPMI1640 media (supplemented with 10% FCS, 2 mM L-glutamine, 100 U/ml penicillin, 100 µg/ml streptomycin) with 1 µg/ml ionomycin, 500 ng/ml PMA and 10 µg/ml Brefeldin A (all Sigma) for 4 hours at 37°C. Following FcR block, cells were surface stained with anti-CD4 PerCP (clone RM4-5), fixed and permeabilised (BD Pharmingen Cytofix/Cytoperm) and then intracellular stained with anti-IFNg APC (clone XMG1.2) and anti-IL-4 PE (clone 11B11). Relevant isotype control stains were included. Alternatively, $CD4^+$ cells were purified from the spleens of naïve BALB/c mice using MACS beads and columns (Miltenyi Biotec), according to the manufacturer's instruction, and stimulated in complete RPMI1640 for 3 days in the presence of 1 µg/ml anti-CD3 (clone 145-2C11) and 0.5 µg/ml anti-CD28 (clone 37.51) with varying amounts of recombinant *Bm*-TPI. Cells were then washed, resuspended in fresh media and stimulated with PMA and ionomycin in the presence of Brefeldin A as above. For *in vivo* transfers of ovalbumin-specific DO11.10 cells, BALB/c mice were injected i.p. with 2×10e6 DO11.10 splenocytes (equivalent to approx 4×10^5 $CD4^+$ cells, data not shown). The next day, mice

were given 0.5×10^6 dendritic cells i.p. pulsed overnight with LPS (100 ng/ml, Sigma) and ovalbumin peptide (pOVA) residues 323–339 (20 μg/ml, Invivogen), then subsequently injected on days 0, 1, 3 and 5 with 100 μg rBm-TPI or PBS control. Control mice were given PBS rather than dendritic cells. At day 7, spleens and peritoneal cells were harvested, stimulated as above and stained with biotin anti-mouse TCR DO11.10 (clone KJ1-26) followed by streptavidin-APC conjugate. Dendritic cells were generated *in vitro* from mouse bone marrow in the presence of GM-CSF [59]. Antibodies were from Biolegend unless stated.

Statistical analysis

Statistical significance was determined using Prism 6 (Graphpad Software Inc.). For comparison between two groups, unpaired Student's *t*-test or Mann-Whitney U-test was used dependent on data normality. Multiple comparisons used one-way ANOVA followed by Tukey's test.

Supporting Information

Figure S1 Human antibodies to Bm-TPI recognise conformational epitopes. IgG1 human ELISA reactivity to "native" or heat-denatured recombinant *Bm*-TPI. Sera is from (A) non-exposed UK residents, (B) endemic normals, (C) microfilaremics, and (D) pathology patients. Only sera producing a positive signal were included in B–D.
(PDF)

Figure S2 Bm-TPI neutralisation impairs embryogenesis. Representative pictures of uterine contents of adult female *B. malayi* parasites recovered from (A) control MOPC31C and (B) 1.11.1 anti-TPI antibody treated mice after 14 days. Note oocytes in control worms with pretzel and stretched Mf, whereas uterine contents in anti-*Bm*-TPI worms are smaller and potentially degraded.
(PDF)

Figure S3 *In vitro* or *in vivo* exposure to *Bm*-TPI does not alter T cell responses. A. *In vitro* production of IL-4, IL-10 and IFN-γ by anti-CD3/anti-CD28 stimulated $CD4^+$ T cells ± *Bm*-TPI. **B**. *In vivo* transfer of OVA-specific DO11.10 transgenic T cells with pOVA-pulsed BMDC ± *Bm*-TPI. (B) Percentage of spleen (top) or peritoneal (bottom) $CD4^+$ that are OVA-specific. **C**. *Ex vivo* production of IFN-γ and IL-4 by gated peritoneal $KJ1\text{-}26^+CD4^+$ cells. Similar results to (B–C) were observed if mice were primed with OVA protein adsorbed to alum adjuvant rather than DC (data not shown).
(PDF)

Author Contributions

Conceived and designed the experiments: JPH RMM. Performed the experiments: JPH DR YH JM LMW SAB. Analyzed the data: JPH JEA RMM. Contributed reagents/materials/analysis tools: JEA AK. Wrote the paper: JPH RMM.

References

1. Babu S, Nutman TB (2012) Immunopathogenesis of lymphatic filarial disease. Semin Immunopathol 34: 847–861.
2. Gregory WF, Atmadja AK, Allen JE, Maizels RM (2000) The *abundant larval transcript 1/2* genes of *Brugia malayi* encode stage-specific candidate vaccine antigens for filariasis. Infect Immun 68: 4174–4179.
3. Babayan SA, Allen JE, Taylor DW (2012) Future prospects and challenges of vaccines against filariasis. Parasite Immunol 34: 243–253
4. Hewitson JP, Grainger JR, Maizels RM (2009) Helminth immunoregulation: the role of parasite secreted proteins in modulating host immunity. Mol Biochem Parasitol 167: 1–11.
5. Johnston MJG, Macdonald JA, McKay DM (2009) Parasitic helminths: a pharmacopeia of anti-inflammatory molecules. Parasitology 136: 125–147.
6. Hewitson JP, Harcus YM, Curwen RS, Dowle AA, Atmadja AK, et al. (2008) The secretome of the filarial parasite, *Brugia malayi* : proteomic profile of adult excretory-secretory products. Mol Biochem Parasitol 160: 8–21.
7. Moreno Y, Geary TG (2008) Stage- and gender-specific proteomic analysis of *Brugia malayi* excretory-secretory products. PLoS Negl Trop Dis 2: e326.
8. Bennuru S, Semnani R, Meng Z, Ribeiro JM, Veenstra TD, et al. (2009) *Brugia malayi* excreted/secreted proteins at the host/parasite Interface: stage- and gender-specific proteomic profiling. PLoS Negl Trop Dis 3: e410.
9. Bennuru S, Meng Z, Ribeiro JM, Semnani RT, Ghedin E, et al. (2011) Stage-specific proteomic expression patterns of the human filarial parasite *Brugia malayi* and its endosymbiont *Wolbachia*. Proc Natl Acad Sci U S A 108: 9649–9654.
10. Knowles JR (1991) To build an enzyme. Philos Trans R Soc Lond B Biol Sci 332: 115–121.
11. Curwen RS, Ashton PD, Sundaralingam S, Wilson RA (2006) Identification of novel proteases and immunomodulators in the secretions of schistosome cercariae that facilitate host entry. Mol Cell Proteomics 5: 835–844.
12. Cass CL, Johnson JR, Califf LL, Xu T, Hernandez HJ, et al. (2007) Proteomic analysis of *Schistosoma mansoni* egg secretions. Mol Biochem Parasitol 155: 84–93.
13. Yatsuda AP, Krijgsveld J, Cornelissen AWCA, Heck AJ, De Vries E (2003) Comprehensive analysis of the secreted proteins of the parasite *Haemonchus contortus* reveals extensive sequence variation and differential immune recognition. J Biol Chem 278: 16941–16951.
14. Liu QY, Corjay M, Feuerstein GZ, Nambi P (2006) Identification and characterization of triosephosphate isomerase that specifically interacts with the integrin alphaIIb cytoplasmic domain. Biochem Pharmacol 72: 551–557.
15. Pereira LA, Bao SN, Barbosa MS, da Silva JL, Felipe MS, et al. (2007) Analysis of the Paracoccidioides brasiliensis triosephosphate isomerase suggests the potential for adhesin function. FEMS Yeast Res 7: 1381–1388.
16. Ikeda R, Saito F, Matsuo M, Kurokawa K, Sekimizu K, et al. (2007) Contribution of the mannan backbone of cryptococcal glucuronoxylomannan and a glycolytic enzyme of *Staphylococcus aureus* to contact-mediated killing of *Cryptococcus neoformans*. J Bacteriol 189: 4815–4826.
17. Furuya H, Ikeda R (2009) Interaction of triosephosphate isomerase from the cell surface of Staphylococcus aureus and alpha-(1→3)-mannooligosaccharides derived from glucuronoxylomannan of Cryptococcus neoformans. Microbiology 155: 2707–2713.
18. Ationu A, Humphries A, Lalloz MR, Arya R, Wild B, et al. (1999) Reversal of metabolic block in glycolysis by enzyme replacement in triosephosphate isomerase-deficient cells. Blood 94: 3193–3198.
19. Schneider AS (2000) Triosephosphate isomerase deficiency: historical perspectives and molecular aspects. Baillieres Best Pract Res Clin Haematol 13: 119–140.
20. Harn DA, Gu W, Oligino LD, Mitsuyama M, Gebremichael A, et al. (1992) A protective monoclonal-antibody specifically recognizes and alters the catalytic activity of schistosome triose-phosphate isomerase. J Immunol 148: 562–567.
21. Zhu Y, Si J, Harn DA, Xu M, Ren J, et al. (2006) Schistosoma japonicum triose-phosphate isomerase plasmid DNA vaccine protects pigs against challenge infection. Parasitology 132: 67–71.
22. Da'dara AA, Li YS, Xiong T, Zhou J, Williams GM, et al. (2008) DNA-based vaccines protect against zoonotic schistosomiasis in water buffalo. Vaccine 26: 3617–3625.
23. Wilson RA, Coulson PS, Betts C, Dowling M-A, Smythies LE (1996) Impaired immunity and altered pulmonary responses in mice with a disrupted interferon-g receptor gene exposed to the irradiated *Schistosoma mansoni* vaccine. Immunology 87: 275–282.
24. Reynolds SR, Dahl CE, Harn DA (1994) T and B cell epitope determination and analysis of multiple antigenic peptides for the *Schistosoma mansoni* experimental vaccine triose-phosphate isomerase. J Immunol 152: 193–200.
25. Reis EA, Mauadi Carmo TA, Athanazio R, Reis MG, Harn DA, Jr. (2008) *Schistosoma mansoni* triose phosphate isomerase peptide MAP4 is able to trigger naive donor immune response towards a type-1 cytokine profile. Scand J Immunol 68: 169–176.
26. McCarthy JS, Wieseman M, Tropea J, Kaslow D, Abraham D, et al. (2002) Onchocerca volvulus glycolytic enzyme fructose-1,6-bisphosphate aldolase as a target for a protective immune response in humans. Infect Immun 70: 851–858.
27. Raverdy S, Zhang Y, Foster J, Carlow CK (2007) Molecular and biochemical characterization of nematode cofactor independent phosphoglycerate mutases. Mol Biochem Parasitol 156: 210–216.
28. Singh AR, Joshi S, Arya R, Kayastha AM, Srivastava KK, et al. (2008) Molecular cloning and characterization of *Brugia malayi* hexokinase. Parasitol Int 57: 354–361.
29. Barrett J, Mendis AH, Butterworth PE (1986) Carbohydrate metabolism in Brugia pahangi (Nematoda: Filarioidea). Int J Parasitol 16: 465–469.
30. Köhler P (1991) The pathways of energy generation in filarial parasites. Parasitol Today 7: 21–25.
31. Tielens AG (1994) Energy generation in parasitic helminths. Parasitol Today 10: 346–352.

32. Ghedin E, Wang S, Spiro D, Caler E, Zhao Q, et al. (2007) Draft genome of the filarial nematode parasite *Brugia malayi*. Science 317: 1756–1760.
33. Banner DW, Bloomer AC, Petsko GA, Phillips DC, Pogson CI, et al. (1975) Structure of chicken muscle triose phosphate isomerase determined crystallographically at 2.5 angstrom resolution using amino acid sequence data. Nature 255: 609–614.
34. Hoffmann WH, Petit G, Schulz-Key H, Taylor DW, Bain O, et al. (2000) *Litomosoides sigmodontis* in mice: reappraisal of an old model for filarial research. Parasitol Today 16: 387–389.
35. Kurniawan A, Yazdanbakhsh M, van Ree R, Aalberse R, Selkirk ME, et al. (1993) Differential expression of IgE and IgG4 specific antibody responses in asymptomatic and chronic human filariasis. J Immunol 150: 3941–3950.
36. Yazdanbakhsh M, Paxton WA, Kruize YCM, Sartono E, Kurniawan A, et al. (1993) T cell responsiveness correlates differentially with antibody isotype levels in clinical and asymptomatic filariasis. J Infect Dis 167: 925–931.
37. Ash LR (1973) Chronic *Brugia pahangi* and *Brugia malayi* infections in *Meriones unguiculatus*. J Parasitol 59: 442–447.
38. McSorley HJ, Harcus YM, Murray J, Taylor MD, Maizels RM (2008) Expansion of Foxp3^{+} regulatory T cells in mice infected with the filarial parasite, *Brugia malayi*. J Immunol 181: 6456–6466.
39. Cortes-Figueroa AA, Perez-Torres A, Salaiza N, Cabrera N, Escalona-Montano A, et al. (2008) A monoclonal antibody that inhibits *Trypanosoma cruzi* growth in vitro and its reaction with intracellular triosephosphate isomerase. Parasitol Res 102: 635–643.
40. Bandi C, McCall JW, Genchi C, Corona S, Venco L, et al. (1999) Effects of tetracycline on the filarial worms Brugia pahangi and Dirofilaria immitis and their bacterial endosymbionts Wolbachia. Int J Parasitol 29: 357–364.
41. Ghedin E, Hailemariam T, DePasse JV, Zhang X, Oksov Y, et al. (2009) Brugia malayi gene expression in response to the targeting of the Wolbachia endosymbiont by tetracycline treatment. PLoS Negl Trop Dis 3: e525.
42. Ziewer S, Hubner MP, Dubben B, Hoffmann WH, Bain O, et al. (2012) Immunization with L. sigmodontis microfilariae reduces peripheral microfilaraemia after challenge infection by inhibition of filarial embryogenesis. PLoS Negl Trop Dis 6: e1558.
43. Denham DA, Medeiros F, Baldwin C, Kumar H, Midwinter ICT, et al. (1992) Repeated infection of cats with *Brugia pahangi* : parasitological observations. Parasitology 104: 415–420.
44. Olivares-Illana V, Rodriguez-Romero A, Becker I, Berzunza M, Garcia J, et al. (2007) Perturbation of the dimer interface of triosephosphate isomerase and its effect on *Trypanosoma cruzi*. PLoS Negl Trop Dis 1: e1.
45. Kuntz DA, Osowski R, Schudok M, Wierenga RK, Muller K, et al. (1992) Inhibition of triosephosphate isomerase from *Trypanosoma brucei* with cyclic hexapeptides. Eur J Biochem 207: 441–447.
46. Singh SK, Maithal K, Balaram H, Balaram P (2001) Synthetic peptides as inactivators of multimeric enzymes: inhibition of *Plasmodium falciparum* triosephosphate isomerase by interface peptides. FEBS Lett 501: 19–23.
47. Enriquez-Flores S, Rodriguez-Romero A, Hernandez-Alcantara G, De la Mora-De la Mora I, Gutierrez-Castrellon P, et al. (2008) Species-specific inhibition of *Giardia lamblia* triosephosphate isomerase by localized perturbation of the homodimer. Mol Biochem Parasitol 157: 179–186.
48. Pearlman E, Hazlett FE, Jr., Boom WH, Kazura JW (1993) Induction of murine T-helper-cell responses to the filarial nematode *Brugia malayi*. Infect Immun 61: 1105–1112.
49. Lawrence RA, Allen JE, Osborne J, Maizels RM (1994) Adult and microfilarial stages of the filarial parasite *Brugia malayi* stimulate contrasting cytokine and immunoglobulin isotype responses in BALB/c mice. J Immunol 153: 1216–1224.
50. Zang XX, Atmadja AK, Gray P, Allen JE, Gray CA, et al. (2000) The serpin secreted by *Brugia malayi* microfilariae, Bm-SPN-2, elicits strong, but short-lived, immune responses in mice and humans. J Immunol 165: 5161–5169.
51. Bellafiore S, Shen Z, Rosso MN, Abad P, Shih P, et al. (2008) Direct identification of the *Meloidogyne incognita* secretome reveals proteins with host cell reprogramming potential. PLoS Pathog 4: e1000192.
52. Gregory WF, Blaxter ML, Maizels RM (1997) Differentially expressed, abundant *trans*-spliced cDNAs from larval *Brugia malayi*. Mol Biochem Parasitol 87: 85–95.
53. Taylor M, Le Goff L, Harris A, Malone E, Allen JE, et al. (2005) Removal of regulatory T cell activity reverses hyporesponsiveness and leads to filarial parasite clearance in vivo. J Immunol 174: 4924–4933.
54. Sartono E, Kruize YCM, Kurniawan A, van der Meide PH, Partono F, et al. (1995) Elevated cellular responses and interferon-γ release after long-term diethylcarbamazine treatment of patients with human lymphatic filariasis. J Infect Dis 171: 1683–1687.
55. McSorley HJ, Grainger JR, Harcus YM, Murray J, Nisbet A, et al. (2010) *daf-7*-related TGF-β homologues from trichostrongyloid nematodes show contrasting life cycle expression patterns. Parasitology 137: 159–171.
56. Lambeir AM, Opperdoes FR, Wierenga RK (1987) Kinetic properties of triose-phosphate isomerase from *Trypanosoma brucei brucei*. A comparison with the rabbit muscle and yeast enzymes. Eur J Biochem 168: 69–74.
57. Kurniawan-Atmadja A, Sartono E, Partono F, Yazdanbakhsh M, Maizels RM (1998) Specificity of predominant IgG4 antibodies to adult and microfilarial stages of *Brugia malayi*. Parasite Immunol 20: 155–162.
58. Hewitson JP, Filbey KJ, Grainger JR, Dowle AA, Pearson M, et al. (2011) *Heligmosomoides polygyrus* elicits a dominant nonprotective antibody response directed at restricted glycan and peptide epitopes. J Immunol 187: 4764–4777.
59. Lutz MB, Kukutsch N, Ogilvie AL, Rossner S, Koch F, et al. (1999) An advanced culture method for generating large quantities of highly pure dendritic cells from mouse bone marrow. J Immunol Methods 223: 77–92.
